# What self-regulation strategies do elementary students utilize while learning online?

**DOI:** 10.1007/s10639-022-11244-9

**Published:** 2022-08-03

**Authors:** Jodie Torrington, Matt Bower, Emma C. Burns

**Affiliations:** School of Education, Macquarie University, North Ryde, NSW 2109 Australia

**Keywords:** Self-Regulated Learning, COVID-19 pandemic, Hypermedia, Online learning, Elementary education

## Abstract

Little is known about the strategies elementary school students use to self-regulate their learning while in a hypermedia environment. This exploratory study investigated the self-regulatory strategies that young students (*N* = 48, *M*_*age*_ = 10.75) utilized while individually completing a 20-min online research task about space. Video data was coded using Azevedo et al.'s (2004) established coding scheme for analyzing self-regulatory behavior in hypermedia environments. Results showed that young students spent the majority of their time using cognitive strategies (*M* = 75.26%) to read and summarise information to complete the task. Little time was taken to plan (*M* = 6.99%) or monitor (*M* = 5.92%) their work or learning processes, which are key attributes of effective self-regulation. The study reveals the disparity between the ability to navigate within a hypermedia environment and utilizing planning and monitoring processes to enhance learning while using digital tools. This study highlights the need for the explicit teaching of planning and monitoring strategies in order for young students to develop the full range of self-regulation skills they need when using technology, for instance while learning from home during COVID-19. Implications for curriculum policy and teacher practice are discussed.

## Introduction

Being self-regulated is an important part of being an effective learner (Zimmerman, 2001). Self-regulated learning (SRL) is defined as the ability to manage thoughts, feelings and actions in order to achieve academic success (Boekaerts, 1999; Pintrich, 2004; Winne & Hadwin, 1998; Zimmerman & Schunk, 2011), and is integral for academic and life-long learning (Dignath & Veenman, 2021). Benefits of improved SRL include increased engagement, achievement, participation and motivation (for overviews, Dignath et al., 2008; Donker et al., 2014; Hattie et al., 1996; Muijs & Bokhove, 2020). Research continues to accentuate the importance of elementary school-aged students developing self-regulatory strategies and highlights the positive impact this ability could have throughout their lives (e.g., Dignath & Veenman, 2021; Muijs & Bokhove, 2020).

Despite extensive research in the area of self-regulation and its confirmed benefits for learning, there is limited research on self-regulation in a technological context, particularly for elementary students. Recent studies have focused on various aspects of SRL such as: digital scaffolds to encourage SRL (Lan et al., 2020; Molenaar et al., 2019), individual SRL strategies in digital contexts (e.g., planning; Chang et al., 2012), creating supportive digital SRL environments (Swalander and Folkesson, 2010), and the SRL strategy use of older students (Azevedo et al., 2013). However, there is a dearth of research that identifies the breadth of SRL strategies that elementary school students apply to their learning, and little to no research focused on computer-based environments. Without a holistic understanding of the varied SRL strategies students use and a detailed understanding of how these strategies are used while learning through technology, it is difficult for teachers to apprehend how they can improve and support students' SRL capabilities.

This study addresses this limited understanding of digital SRL behavior by examining the SRL strategies used by 48 elementary students (*M*_*age*_ = 10.75) as they complete a research task in a hypermedia environment. Hypermedia environments are defined as web-based sites that are open-ended, non-linear in structure, and incorporate a range of multimedia including text, images, videos, hyperlinks, and animations, and are complex for young students to navigate (Azevedo et al., 2013). While much educational research focuses on outcomes of interventions, this study aims to help educators understand the *processes* by which their students learn. This observational exploratory study provides insight into the SRL strategies employed by elementary students, and the extent to which they used them, while they were learning in a hypermedia environment. A lack of detailed understanding of how elementary students actually self-regulate while undertaking digital tasks limits the progress we can make in this area: it is not possible for teachers to design evidence-based scaffolds for their students, or software developers to create effective supports for learners in their programs, or teacher educators to design impactful professional learning for teachers, or indeed for researchers to deeply understand the self-regulatory processes that elementary students do (and don't) undertake so that we can identify important areas for further research. This study addresses this need by answering the question: What self-regulation strategies do elementary students apply, and to what extent, when using technology?

## Literature review

### Self-Regulation for Learning (SRL)

The field of Self-Regulated Learning (SRL), which draws on educational psychology theories and research, is well-established. The field examines how learners use their initiative, persevere with a task, and adapt their skills in order to pursue their learning goals (Zimmerman, 2009). Self-regulation theorists assume that students can be (meta)cognitively, motivationally, emotionally, and behaviorally active participants in their own learning. Across the diversity of theoretical perspectives (e.g., Pintrich, 2000; Winne, 2001; Winne & Hadwin, 1998; Winne & Perry, 2000; Zimmerman, 2000, 2001), there are two common features in SRL models that define self-regulated learners: 1) they monitor the effectiveness of their strategy use during learning and respond to this self-oriented feedback accordingly; and 2) they can verbally justify the self-regulated strategy or process they are using (for synthesis see Zimmerman & Schunk, 2011).

Self-regulation is associated with a host of positive outcomes for students (Dignath & Veenman, 2021; Zimmerman & Schunk, 2011). There are numerous studies that have investigated specific areas of self-regulation within the typical face-to-face classroom context that have suggested a causal relationship between self-regulation strategy use and academic performance (for meta-analyses see Hattie et al., 1996; Dignath et al., 2008; Donker et al., 2014). It has been identified that effective SRL intervention programs need to be connected with specific content and be supported by the teaching–learning context to encourage metacognition and self-regulation. Additionally, even though students (including of elementary age) are developmentally capable of becoming self-regulated (Pekrun, 2020; Zimmerman, 2011), it is crucial for students to be explicitly taught learning strategies as it cannot be presumed that self-regulatory strategies are spontaneously acquired (Dignath & Veenman, 2021; Dignath et al., 2008; Donker et al., 2014; Hattie et al., 1996; Muijs & Bokhove, 2020). Despite research highlighting the importance of direct teaching, there is a surprising lack of research about the variety of SRL strategies that students, particularly elementary students, employ. Such research is needed to help teachers understand which strategies students naturally apply and which strategies teachers need to explicitly teach.

### SRL in computer-based learning environments

Research of SRL in digital contexts has also indicated that SRL strategies need to be explicitly taught in these environments (Azevedo et al., 2013; Ferrer-Esteban, 2016; Uhls, 2012; Yot-Domínguez and Marcelo, 2017). Comprehensive research focusing on late high school and university students over the past two decades by Azevedo and colleagues shows that self-regulation is challenging for older learners in computer-based learning environments. This is due to both the complex nature of online environments and the self-regulatory demands of managing this complexity (Azevedo & Cromley, 2004; Azevedo et al., 2004; Azevedo, 2005; Azevedo et al., 2010; Azevedo et al., 2013). They have found that high school and university students do not always use planning, monitoring and reflection strategies while learning through technology, and argue that this limits the effectiveness with which they learn in the computer-based learning environment (Azevedo, 2005; Azevedo et al., 2013). However, it is not yet known whether these findings also apply to younger learners and whether the absence of other strategies may impede the learning of younger learners.

Researchers have focused on embedding prompts and scaffolds within digital programs to stimulate self-regulatory behavior (see Zheng, 2016; Azevedo et al., 2013; Greene et al., 2011), because software and learning platforms are not generally embedded with obvious self-regulatory prompts (Azevedo et al., 2004; Azevedo, 2005). Greene and colleagues (2011) concluded however, that even if scaffolds are provided, it is often the lack of independent self-regulation skills, knowledge or motivation of students that cause their failure to benefit in this structured digital environment. Research in educational technology continues to concentrate on developing effective digital SRL programs, *rather* than helping students to learn independent self-regulation capabilities. Although prompts and scaffolds are beneficial to users (Azevedo et al., 2010), issues remain. Specifically designed programs to promote SRL strategy use (see Azevedo et al., 2009, 2013) are not widely available, making these programs unlikely to be known and applied in the majority of schools. Moreover, specific programs are facilitated through the technology. Given disparities in school technological resourcing (OECD, 2020) and the variety in types of technology available to students, not all schools will have access to specific SRL software, and in many cases these programs may not be compatible with the available technology. Taken together, this highlights potential issues with developing program-based solutions before understanding the ways in which students are self-regulating in hypermedia environments. It is necessary for researchers and educators to understand *how* students independently self-regulate with the technology they have, without needing to rely on external programs. Indeed, researchers have argued that more focus should be placed on explicitly teaching students what self-regulation means, *why* it is important for their learning and *how* to apply it in a digital context (Azevedo et al., 2013; Donker et al., 2014; Greene et al., 2011; Yot-Domínguez and Marcelo, 2017). However, despite this general consensus, very little research has identified the ways in which all learners – and apparently none related to elementary learners specifically—self-regulate in hypermedia learning environments.

The findings of past research, exacerbated by the COVID-19 pandemic/ remote learning era, further highlight a critical gap in the research: a lack of understanding about the extent to which (and how) elementary school-aged learners are self-regulating when using technology. This research is needed to establish the SRL strategies that are understood and applied by elementary students in hypermedia environments, and most importantly, the ones that are overlooked or used briefly. Educators, and digital SRL program developers, will be better informed if current SRL strategy use is measured and analyzed. This study explored the self-regulation strategies used by elementary students in hypermedia environments to address this gap and provide a baseline for future research and development.

## Methodology

This qualitative study, with supplementary quantitative analysis, investigated the self-regulatory strategies that elementary students used during a 20-min research task using an online hypermedia environment. Quantitative data detailing the type of strategy and amount of time spent using each strategy was coded and analyzed before being synthesized with qualitative researcher observations and transcriptions of student discourse from the 'talk-aloud' protocol (Kistner et al., 2010) which required students to explain their thought process and decisions aloud while they completed the task. Thus, various viewpoints of actual student behavior (what they were doing), and thinking (what they said) could be triangulated for increased validity (Creswell and Creswell, 2017; Greene, 2007; Johnson, 2017).

### Participants

A K-12 metropolitan coeducational school in a capital Australian city was purposely selected for this study, based on an established previous cooperative research relationship as well as the 1:1 student to mobile technology ratio applied at the school. Because of this, the generalizability of this exploratory research is considered in the limitations section of this paper. The Index of Community Socio-Educational Advantage (ICSEA) for the school is 1101, higher than the national average of 1000. Of the total student population of 1080, 2% identify as Indigenous and 7% have a language background other than English. The ICSEA identifies 44% of students as being in the top quarter of the scale of socio-educational advantage, which is considerably higher than the average Australian distribution of 25%.

Three Year 5 and three Year 6 classes (*M*_*age*_ = 10.75) were selected because they had used personal mobile devices (iPads) regularly for the previous 3 years. Choosing a sample of students who were confident in the use of technology meant that the study could focus on how elementary school students self-regulate their learning when using technology, without substantial interference caused by students being unable to effectively use the technology itself. This served to ensure that the findings of the study were not confounded by students’ technological ability and are positioned to disentangle effective self-regulatory strategies with technology.

Of the possible Year 5 and 6 students (*N* = 150), 83 were granted permission by their parents/guardians to participate in the study (55% response rate). Due to time constraints and to reduce administrative burden on the school and teachers, of the 83 study participants, 8 students were randomly chosen from each class to form the final sample for this study (*n* = 48; 50% female). The reasons for, and limitations of this smaller sample size are unpacked in the Discussion section of the paper; however, it is important to note that previous well-validated studies that have taken similar methodological approaches have had similar sample sizes for the depth of analysis being performed (Azevedo et al., 2003, 2007). Thus, the sample size is considered sufficiently large and appropriate for the analyses that are conducted. Observations were conducted during Term 1, 2021 while the school was operating normally without any COVID-19 interruptions or restrictions. All students were attending class with their regular teacher, and no disruptions due to school closures had occurred in the school year either before or during the observational period.

### Research task and procedure

Selected participants completed a short online research-based task organized and prepared by the researchers. The topic of 'Space' was selected as the holistic theme of the research task. Students were asked to choose their topic of interest from six provided sub-topics: The Planets, Stars, The Sun, The Moon, Black Holes and Space Exploration. Students were asked to research the topic and draft a paragraph that could teach their peers something about their chosen topic. Students were given 20 min to complete the task.

The hypermedia environment, *Britannica School* (Encyclopedia Britannica, 2021), included text, videos, photos, videos and animations. For the research component of the task, each sub-topic was accompanied by a link to the school's Britannica School website, although students were also permitted to use the Internet. During the task, students were allowed to use all the features incorporated in the *Britannica School* site such as the search function, hyperlinks, the range of multimedia incorporated on the page, and the ability to change the Lexile level of difficulty to easier or harder. There are three difficulty levels within the database (low, middle high); the 'Middle' level was chosen as a starting point for student research. Participants were familiar with the database and how to access it because it is used regularly in classroom activities.

The task for students was designed to be open-ended, and desirably difficult, such that it required self-regulation strategies to be used. Specifically, students had choice of topic, process, resources and the structure and length of the written summarization. Students could also leverage the functionality of the digital devices they were using. All of these independent tasks require self-regulation strategies to be utilized (e.g. planning, monitoring) (Azevedo, 2013).

Prior to students beginning the task, a detailed script was read to, and clarified with, each participant so that task expectations were clear. This included outlining the expectation of the written portion of the task, as well as ensuring they understood how to access photos, videos and related links. Additionally, a detailed explanation of the 'talk-aloud' protocol was given (Kistner et al., 2010). Students were also given time to identify and set-up their choice of program on their mobile device to record their research notes and writing. Students were then allowed to complete the task individually in a quiet breakout space, under the supervision of the lead researcher. Each student’s writing was collected at the end of the 20 min.

### Data collection

SRL can be considered an event and therefore attempts can be made to measure it during learning in computer-based learning environments (Azevedo et al, 2010). In order to better understand the relationships and interaction between technology and self-regulation, Azevedo et al. (2004) developed an inventory of 33 processes that could be utilised while observing students' completing tasks using technology. Their work synthesised several key SRL models (Pintrich, 2000; Winne, 2001; Winne & Hadwin, 1998; Winne & Perry, 2000; Zimmerman, 2000, 2001) and outlined five major areas of self-regulation to measure while learning in a digital environment:

1. Planning processes (including goal setting and prior knowledge activation);
2. Monitoring processes;
3. Cognitive strategy use during the learning;
4. Processes for handling task difficulties and demands; and
5. Interest (motivation)

Azevedo et al. (2004) argue that these 33 processes, grouped in the above five areas, are key self-regulatory strategies needed when using a computer-based learning environment and reflect the major phases of self-regulation. Following Azevedo's methodological approach, two online measures (captured *during* the actual performance task) were used during the student observations: 1) student self-reporting using a 'talk aloud' protocol (Kistner et al., 2010); and 2) observation by the lead researcher of the student completing the task using Azevedo, Cromley and Seibert's coding scheme (2004). The observational sessions were video recorded for transcription and analysis.

### Data analysis

#### Coding student videos

Student observation videos were initially uploaded to iMovie, and trimmed to 20:00 min exactly (from the end of each video) before being imported to the video analysis software, *V-Note* (version 3.1.0). As the raw data from *V-Note* compares time spent on strategies with the overall video length, the total time of the video was standardised for consistency. This software program was chosen for three reasons: (1) event sampling ability, where observed behaviors can be precisely time-sampled to show accurate temporal flow between activities and behaviors; (2) capacity to generate time correlations with raw data; and (3) ability to attach transcriptions to the identified code (Mangold, 2018). Video recordings were considered crucial for elementary school observations, where the 'talk-aloud' protocol could supplemented and contextualised. Such an approach also enables us to account for individual differences between students and how much they shared during the ‘talk-aloud’. Video allowed the exact start and end time of the behaviors to be recorded accurately.

The coding scheme of Azevedo et al. (2004), as previously identified, was used to analyze student behavior. This framework included five main categories of *Planning*, *Monitoring*, *Strategy Use*, *Processes for Handling Task Difficulties and Demands*, and *Interest*. Each category included sub-variables that provided specific clarification of each phase and include key processes relevant to hypermedia environments. The framework included four planning variables, six monitoring variables, seventeen strategies, five task difficulty and demands and one interest variable (Azevedo et al., 2004; see Appendix A for full coding framework).

Initial video analysis indicated the requirement to include two additional coding points in the *Strategy Use* category that were not addressed in the 2004 framework: 1) *watching video for information*; and 2) *writing own knowledge without any reference to hypermedia environment*. This was due to the original framework acknowledging that participants *selected* a 'new informational source' but the only option for time spent on acquiring information was when they '*read* new paragraph'. When students were typing information for their research paragraph without any access of, or reference to the hypermedia environment, this was doubled-coded as both *prior knowledge activation* (*Planning*) (as per the original framework specification) as well as *Typing Using Prior Knowledge—No Hypermedia* (*Strategy Use*). We wanted to be able to distinguish that participants were typing their information and using this *strategy* as a research technique. The addition of these subcategories can be considered a methodological contribution to the Azevedo framework and its application in contemporary elementary school settings.

Videos were coded using all 35 codes. A codebook was maintained throughout the study to uphold reliable coding over time. The original definitions for each coding category were retained and new examples drawn from the student videos were added (Appendix A). This process clarified the coding framework, updated the relevancy to relate to elementary school participants and ensured consistency and shared understanding between researchers and the second rater. Every utterance and behavior of student and researcher was coded. The researcher directly prompted each participant at the start of the task with two questions: 1) *"Why did you choose [black holes]?"*; and 2) *"So, how do you start on a task like this?"*. These two questions provided a means of initiating conversation, established rapport and encouraged the 'talk-aloud' protocol at the start of the task. Statistical raw data of frequency, duration and percentages of codes over each 20-min video was exported from *V-Note* to Excel and Statistical Package for the Social Sciences (SPSS) Version 28 for analysis. The total time distribution exceeded 20 min for 8 out of 48 observations, which were subsequently standardised to 20 min. The main reason for this overshoot of time was the double-coding of the newly added code *Typing Using Prior Knowledge—No Hypermedia* (*Strategy Use*) with *Prior Knowledge Activation* (*Planning*), the original coding protocol. For the 8 students, this caused their total time distribution to be an average of 4.84% (58 s) over the 20-min task time given. Standardisation of these 8 observations to 20 min of coding in total allowed for calibrated comparison between all participants.

Two series of paired sample *t*-tests were performed. The first was used to determine whether there were significant differences between main categories of *Planning*, *Monitoring*, *Strategy Use*, and *Processes for Handling Task Demands and Difficulties*. The second series of paired sample *t*-tests was used to examine the differences between subcategories within each main category. Pair samples* t*-tests were determined to be the most appropriate because the self-regulation variables do not reflect independent groups, distinct conditions, or repeated measures (i.e., collected across time). Rather, each (sub)category of self-regulation is a distinct variable for which each student has cross-sectional data.

#### Inter-rater agreement

Inter-rater agreement was established by training a research colleague about the five main categories of the coding framework. Initially, one video with selected segments was coded jointly, in order to clarify misunderstandings. A further ten videos were randomly chosen and ten coded segments were designated within each video, totalling 100 segments that were independently coded. There was an agreement on 97 out of 100 segments, yielding an inter-rater agreement of 0.97. Inconsistencies were resolved on discussion, as the three disputed segments were misinterpreted due to the segment being out of context. For example, this made it challenging to decide whether a student editing written work was coded as copy/pasting (*Task Demands and Difficulties*) or reviewing work (*Strategy Use*). The level of inter-rater reliability was deemed high and acceptable, enabling analysis of data to proceed with confidence.

## Results

### What self-regulation learning strategies do students apply, and to what extent, when using technology?

The descriptive statistics of the 35 codes are reported in Table 1. Means (percentage of total task time/ number of seconds) and standard deviations for each main category and sub-categories are outlined, as well as the number of students observed using each category.

**Table 1:** Means, standard deviations and number of students observed using each category for the 35 coding categories

Category	Sub-category	*M (% of time out of 20 min)*	*M (in seconds)*	*SD*	Number of students (out of 48)
1) PLANNING	- Planning	2.58	30.96	1.89	48
	- Goal setting	.75	9.00	1.19	21
	- Prior knowledge activation	3.61	43.32	9.31	30
	- Recycle goal in working memory	.36	4.32	.13	4
2) MONITORING	- judgement of learning	.99	11.88	1.33	32
	- feeling of knowing	.30	3.60	.59	16
	- self-questioning	.77	9.24	1.31	25
	- content evaluation	1.61	19.32	1.84	40
	-identify adequacy of information	1.31	15.72	1.73	31
	- monitor progress towards goals	.94	11.28	1.56	28
3) STRATEGY USE	- selecting new informational source	3.67	44.04	5.68	35
	- coordinating informational sources	.01	.12	.05	1
	- read new paragraph	20.02	240.24	11.78	48
	- review notes	4.55	54.60	6.13	39
	- memorization	.05	.60	.26	2
	- free search	.11	1.32	.58	2
	- goal-directed search	3.24	38.88	6.70	19
	- summarization	31.80	381.6	12.97	48
	- taking notes	.50	6.00	2.43	3
	- drawing	.37	4.44	2.55	1
	- re-reading	4.82	57.84	5.28	37
	- inferences	.93	11.16	2.90	23
	- hypothesizing	.32	3.84	.90	10
	- knowledge elaboration	.39	4.68	.89	13
	- mnemonic	.00	0	.00	0
	- evaluate content as answer to goal	.03	.36	.17	2
	- find location in environment	.09	1.08	.26	6
	**(new) watching video for information	4.90	58.80	10.05	13
	**(new) typing using prior knowledge no hypermedia	2.93	35.16	9.01	11
4) PROCESSES FOR HANDLING TASK DIFFICULTIES AND DEMANDS	- time and effort planning	.29	3.48	.96	11
	- help-seeking behavior	.18	2.16	.42	15
	- task difficulty	.09	1.08	.40	3
	- control of context	4.69	56.28	4.82	43
	- expectation of adequacy of information	.15	1.80	.39	8
5) INTEREST	- interest statement	1.17	14.04	1.21	42

**Fig. 1:**
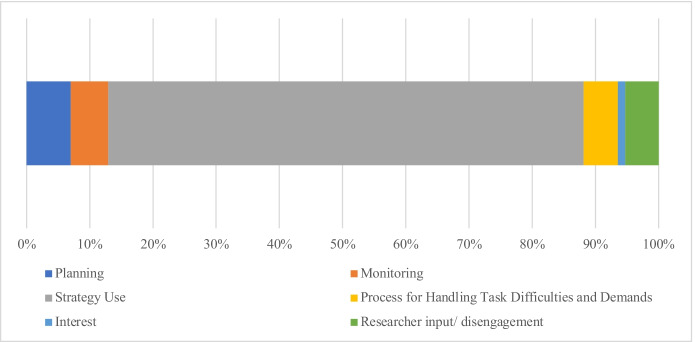
Mean time distribution of the main categories over the 20-min task duration

To address the research question, the mean percentage of time (of the total 20 min video) and standard deviation for each of the five main categories were calculated: *Planning* (*M* = 6.99% [83.88 s], *SD* = 9.44); *Monitoring* (*M* = 5.92% [71.04 s], *SD* = 3.93); *Strategy Use* (*M* = 75.26% [903.12 s], *SD* = 12.23); *Processes for handling task difficulties and demands* (*M* = 5.39% [64.68 s], *SD* = 5.22); and *Interest* (*M* = 1.17% [14.04 s], *SD* = 1.21). Remaining time was either accounted for by the researcher asking a question to prompt students, or students not engaging with the hypermedia environment (*M* = 5.27% [63.24 s]) (Fig. 1).

**Fig. 2:**
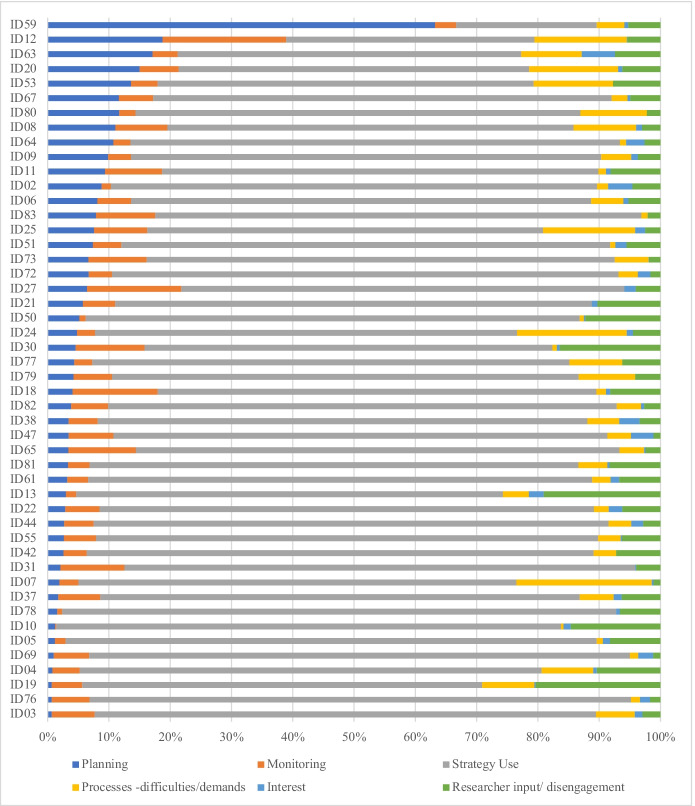
Time distribution for the main categories for each participant, ordered by time spent planning

This showed that students mainly used cognitive strategies throughout the online task and they spent slightly more time planning than monitoring. Paired sample *t*-tests were used to determine if there was a significant difference in time spent between the five main categories A Bonferroni corrected *p*-value of 0.005 was applied during calculations to account for the 10 tests being conducted. Results revealed significant differences between the time spent on Strategy Use with all other categories, for example Planning/Strategy Use: *t*(47) = -22.969, *p* < 0.001. Interest was also significantly different to all other main categories (Table 2). The time distribution for all observations for the five main categories, ordered by Planning, is shown in Fig. 2.

**Table 2:** Paired sample t-test results for the five main categories

	Planning	Monitoring	Strategy Use	Process Demands/ Difficulties	Interest
Planning	-				
Monitoring	.737	-			
Strategy Use	-22.969***	-34.475***	-		
Process Demands/ Difficulties	1.093	.575	31.429***	-	
Interest	4.233***	7.628***	42.154***	5.291***	-

For all *t*-tests, degrees of freedom = 47

^*^p < .05, **p < .01, ***p < .001

*Ex-post facto* analysis found no significant differences when investigating both 'year level' and 'gender' between the time spent on the five main categories.

### Sub-category findings for planning, monitoring, strategy use and processes for handling task difficulties and demands

#### Planning

The four variables are presented in Table 3, grouped by mean similarity. Students spent the most time in this category *activating prior knowledge* (*M* = 3.61%), whether it be orally acknowledging prior understandings (for example, talking about constellations and realising *"I can see [the saucepan] from my house too"* ID67) or searching their memory for previous knowledge (*"I'm writing about what I've heard about Black Holes"* ID50). One student spent 61.8% of the time typing previously learned knowledge without engaging with the hypermedia environment, which likely inflated the results for this sub-category. *Planning* (their approach to the task) (*M* = 2.58%), was the next highest sub-category, which could be attributed to the direct prompting of the researcher at the start of the task. When asked about how they would start, students were able to verbalise their approach (for example *"I first look at a lot of, like, websites and see what's similar, so I know it's reliable, so that I'm not telling, like fake stuff. And, like, I would go to the library [online catalogue] and search it up on there so I can get, like lots of websites"* ID44).

**Table 3:** Planning Variable Means and Standard Deviations in descending order

	Variable	*M*	*SD*
Tier 1	Activating prior knowledge	3.61	9.31
	Planning (their approach to the task)	2.58	1.89
Tier 2	Goal setting	.75	1.19
	Recycle goal in working memory	.36	.13

Results showed that students spent an average of 31 s out of 20 min *planning* and only 9 s *goal setting* (*M* = 0.75%). Over half the students (27 of 48) spent no time goal setting. Students who demonstrated *goal setting* verbalised specific knowledge they wanted to research, usually after a time of initial reading using the hypermedia environment (for example, *[after 3 min reading] "I'm gonna start my blurb by who was the first person [in space] and how he travelled and what he travelled in"* ID27)*.* Only two students wrote their goals down.

Only four participants *recycled the goal in working memory* (*M* = 0.04%), and this strategy was used significantly less than any other planning strategy. Overall, students spent significantly more time *planning* than *goal setting*, *t*(47) = 6.571, *p* < 0.001, and significantly less time *goal setting* than *activating prior knowledge t*(47) = -2.089, *p* < 0.001 (Appendix B).

#### Monitoring

For the six monitoring sub-categories, results indicated that students spent the most time making judgements about the content (*content evaluation*: *M* = 1.61%, 19 s; *identifying the adequacy of information*: *M* = 1.31%, 15.8 s), rather than judgements about one's own learning processes (*judgement of learning: M* = 0.99%, *monitor progress towards goals: M* = 0.94%, *self-questioning: M* = 0.77%, *feeling of knowing: M* = 0.30%) (Table 4).

**Table 4:** Monitoring Variable Means and Standard Deviations in descending order

	Variable	*M*	*SD*
Tier 1	Content evaluation	1.61	1.84
	Identifying the adequacy of information	1.31	1.73
Tier 2	Judgement of learning	.99	1.33
	Monitor progress towards goals	.94	1.56
	Self-questioning	.77	1.31
	Feeling of knowing	.30	.59

Over 83% of students were able to reflect on, and make decisions about the content relative to their topic (*content evaluation*) and verbalise this (for example, *"Now I realise I should probably name the ship he came in, which was the Apollo 11 I think."* ID27). Students made decisions using all types of content in the hypermedia environment, for example while reading text: "*Now I'm just going through, like, little bits that catch my eye, like things that seem to have a lot of stuff that could be very important to write down"* ID65; while using images: "*I just want to see a visual, so that I can….comprehend what they're talking about"* ID83; or after watching a video: *"OK, I'll write that in"* ID69. Two-thirds (65%) of students were able to assess the usefulness or adequacy of the content holistically (*identifying the adequacy of information*), verbalising their decision (for example, *"Um, I don't know whether to trust this website [Wikipedia]… it isn't seem to be saying what it's….really…is"* and another website as acceptable *"I think because it's a school website, it's made for kids to understand it"* ID37).

Of the sub-categories focused on learning processes, one-third of students (16 of 48) spent no time making a *judgement of learning*, that is, monitoring whether they understood, and were processing the content effectively. Students who used this strategy made comments such as *"That paragraph is a little bit too hard for me to comprehend"* (ID76), *"OK, that's confusing"* (ID09) and *"I wanna make it Level 1 [reading] so then it's easier"* (ID11). Just over half (58%) of the students reflected on whether they had achieved their goal or sub-goals (*monitor progress towards goals*) typically after reviewing their written paragraph, for example: "*Now, I think I've probably done 5–6 sentences, so I'm going to read it and see if it all makes sense and edit and stuff"* (ID27) and "*I've written three sentences now, and now I think it's enough"* (ID11). Only 25 (of 48) posed questions to themselves in order to monitor and improve their understanding (*self-questioning*). Questions were asked while engaging with the hypermedia environment (for example, *[while viewing a photo of a black hole] "Dunno why they're orange?"* ID37; or *[reading through information] "OK, should I use that?"* ID69) as well as while typing their paragraph (for example, *[about stars] "I think they're made of gas?* ID09). Students on average spent 3.6 s out of 20 min (*M* = 0.30%) using the *feeling of knowing* strategy, although 32 of the 48 students did not demonstrate this behavior. This activation of long-term schema, where one is aware of information from the past yet cannot fully recall it, was evident by comments such as *"I'm pretty sure, I'm pretty sure, it's from scientists instead but…um…but yeah"* (ID53) and *[while typing prior knowledge] "I can't remember it"* (ID67).

Paired sample *t*-tests (Appendix B) revealed significant differences between *feeling of knowing* and all other variables in this category. *Self-questioning* and *content evaluation* revealed a significant difference, *t*(47) = -2.405, p < 0.001, with students spending significantly more time evaluating whether the content was suitable than posing questions to evaluate their own learning.

#### Strategy use

The means, standard deviations, time spent and number of students using each strategy for the 19 variables in this category are presented in Table 1. To establish whether strategy means were significantly different from zero, one-sample *t*-tests were performed. This confirmed that 12 out of 19 strategies were significantly different from zero (Table 5). The remaining 7 variables: co-ordinate information sources, taking notes, drawing, free search, memorization, evaluate content as answer to goal and mnemonic were therefore not included in subsequent analysis as they were rarely used by students.

**Table 5:** Strategy Use Variable Means and Standard Deviations in descending order

	Variable	*M*	*SD*
Tier 1	Summarizing	31.80	12.97
	Read new paragraph	20.02	11.78
Tier 2	Watching video for information	4.90	10.05
	Re-reading	4.82	5.28
	Review notes	4.55	6.13
	Selecting new information source	3.67	5.68
	Goal-directed search	3.24	6.70
	Typing using prior knowledge no hypermedia	2.93	9.01
Tier 3	Inferences	.93	2.90
	Knowledge elaboration	.39	.89
	Hypothesizing	.32	.90
	Find location in environment	.09	.26

The remaining 12 variables are presented in three tiers in Table 5, grouped by mean similarity. All students spent over half their task time *summarizing* and *reading new paragraphs*, ranking the highest of the variables (Tier 1). Students were able to explain how they summarized information and the importance of avoiding plagiarizing, indicating explicit knowledge and understanding of this strategy, for example *"So, whenever I read an article and I finish, I try to think of how I can make it my own words, so I don't copy it"* (ID27) and *"I don't copy the exact thing because that's plagiarizing, so, you have to change it up a little bit"* (ID76). As the task was research focused, *reading new paragraphs* was a common strategy and if it wasn't directly stated by the student (for example: *"Now I'm gonna read a little bit more of the article, because I finished my first two sentences"* ID27), it was obvious by their scrolling behavior and navigation through the text.

The following six variables had considerably lower means of a similar range, grouped as Tier 2 (Table 5). In this tier, *re-reading, reviewing notes and selecting new information sources* were commonly used strategies used by 37, 39 and 35 students respectively. This indicated that students knew to clarify their understanding of information by revisiting it (*"I'm going back 'cause I can't remember what the fact was"* ID24), and were aware of the need to read over and edit their written work (*"Now, I'm just gonna go over what I've already got—see if everything written just makes sense"* ID76). Students demonstrated they could confidently select the multimedia options available on the hypermedia page, whether this meant viewing videos, animations, images, text or other websites (*"I'm going to watch this video"* ID02). Many students (19) used *goal-directed searches*, searching the online environment with a specific purpose. Students were confident using this strategy, for example: "*I'm* *gonna* *search up how many Black Holes the scientists think there is in the Milky Way"* (ID76). *Watching video for information* was the highest mean in Tier 2, yet the strategy was only used by 13 students. When asked why they chose to watch a video, most students acknowledged that they found it easier to watch and listen rather than read (for example: *"I watch videos. It's hard for me to read."* (ID19) or *"I watch videos. It's quicker."* (ID65). Not all students realised they had the freedom to pause the video as needed until it was pointed out by the lead researcher. Finally, 11 students spent time just typing their own prior knowledge, without interacting with the hypermedia environment as required. (*"Some of this stuff I've read, some of this stuff I've known, like the meteorites."* ID51). One student (ID59) spent 13 of the 20-min observation using this strategy.

The mean results for the remaining four strategies (Tier 3) were < 1% (Table 5). Almost half the students (23) demonstrated *inferences* based on what they had read or watched, however the time spent on this was typically brief, for example: *"I'm thinking that the black hole must be deep* *'cause* *it says, um, 'infinite'*" (ID22). Elaboration on what was just read, using prior knowledge (*knowledge elaboration)*, was demonstrated by 13 students, for example: *"I'm still shocked at how a black hole can suck the matter out of something. Like, a black hole could suck this (holds up instruction sheet) into matter. Like this is solid!"* (ID 13). Only 10 students *hypothesized* by asking questions that went beyond what they had read or watched, for example: *"I'm just thinking [about what they’d just read about black holes]: What happens if a human goes near it? Or some kind of spacecraft?"* (ID37). Making inferences, hypothesizing and elaborating are strategies that involve reflecting on information and spending time to explore the information in more depth. These strategies were only demonstrated by a small group of participants.

#### Processes for handling task difficulties and demands

Paired sample *t*-tests revealed significant differences between *control of context* and all other variables in this category due to its considerably higher mean (*M* = 4.69%, 56.3 s) (Appendix 2). Results indicate that students spent significantly more time controlling their digital context than the other variables, enhancing the reading, viewing, and organising of their digital environment. Conversely, only three students expressed *task difficulty* (*M* = 0.09%) during the task. There was little awareness from students about time management (*time and effort planning*, M = 0.29%) with only 11 students inquiring about the amount of time remaining during the task (Table 6).

**Table 6:** Processes for Handling Task Difficulties and Demands Variable Means and Standard Deviations in descending order

	Variable	*M*	*SD*
Tier 1	Control of context	4.69	4.82
Tier 2	Time and effort planning	.29	.96
	Help-seeking behavior	.18	.42
	Expectation of adequacy of information	.15	.39
	Task difficulty	.09	.40

Students were able to use features of the hypermedia environment to enhance the reading and viewing of information (*control of context*). They were confident with their digital capability and demonstrated skills such as manipulating the keyboard of an iPad to split or move as needed, adding emojis to written work, split or overlaid screen, taking screenshots, manipulating photos, using drawing tools, zooming to enhance viewability and navigating videos.

#### Interest

Only brief statements of interest were observed (*M* = 1.17%, 14.04 s) by most students (42 out of 48). Typically, students were reacting to new information gained, for example: *"Oh! That's interesting! That light can't even escape from Black Holes!"* (ID76), or responding to being asked why they chose the particular topic, for example: *"It sounded interesting, and I haven't learned about them before"* (ID37). Sometimes, students made the judgement of whether the information would be interesting for their audience to read before spending time summarizing it, for example: *"I chose to write this because it is an interesting fact, and it might 'wow' people"* (ID02). The students were cooperative participants and were focused on completing the task.

## Discussion

The results of this study shed new light on the self-regulation processes that elementary students apply when learning in computer-based learning environments. Our results show that when these young students use an online hypermedia environment to complete a short research task without any scaffolds or prompting to use self-regulatory processes, they spend on average 75% of their time using cognitive strategies, and comparatively less time planning (7%), monitoring (6%), managing task difficulties or demands (5%) or indicating interest (1%). The fact that they spent significantly longer using cognitive strategies to complete the task (such as *reading, re-reading, summarizing and reviewing*) rather than on other key processes, suggests that elementary students do not have an intuitive understanding of the range SRL strategies needed in computer-based learning environments. This indicates elementary school students need more direct support in applying the SRL strategies that have been identified as most effective. This finding is consistent with previous research of university undergraduate participants that indicated that when self-regulatory scaffolding was not provided in hypermedia environments, or if students had not been specifically taught about self-regulatory processes to use in a hypermedia environment, learning was likely to be negatively impacted due to their lack of planning, monitoring or reflection (Azevedo et al., 2013). It also aligns with research that argue that students of all ages have difficulty regulating their learning when using a hypermedia environment (Azevedo & Cromley, 2004; Azevedo et al., 2004; Azevedo, 2005; Moos & Azevedo, 2006). This study therefore extends Azevedo et al.'s (2004) framework by demonstrating it can be applied to younger students and provides further evidence for the importance of understanding SRL behavior holistically through the use of the five identified SRL categories.

Our study shows that monitoring strategies are not necessarily instinctive for young students. The limited time that students spent applying monitoring strategies raises that possibility that the strategies were unknown. The observed lack of monitoring strategy application is concerning, given the wealth of research recognizing the impact and benefits of explicit metacognitive instruction for students, particularly on academic performance (Dignath & Veenman, 2021; Dignath et al., 2008; Donker et al., 2014; Hattie et al., 1996). Research has shown that more successful students in hypermedia environments regulate their learning using significantly more monitoring processes and strategies than their peers (Azevedo, 2005). Current research continues to reiterate the necessity of teaching metacognitive strategies to students (Dignath & Veenman, 2021; Muijs & Bokhove, 2020), yet the knowledge and verbalization of these strategies from students in this study was limited, indicating that perhaps this importance has not yet been fully realised in schools. It has been shown that teachers can provide effective scaffolding of self-regulatory strategies to students (Azevedo, 2005), however they would need to be aware of what these strategies were in order to explicitly teach them.

A logical explanation for the lack of monitoring skills exhibited by students in our study is that teachers have sparse guidance from official curriculum documents about monitoring processes in teaching practice. For example, the Australian Curriculum (ACARA) (Australian Curriculum Assessment and Reporting Authority, 2021) promotes dispositional thinking pedagogy (Churchill, 2019), of which reflection (metacognitive thinking) is a key component, however there is no explanation of current self-regulation findings, or specific monitoring examples and strategies for teachers to implement. Australia's curriculum is not unique in its lack of metacognitive monitoring support. The British and American elementary curricula (National Curriculum in England: Primary Curriculum, 2021; Common Core State Standards Initiative, 2021) also fail to provide detailed support for teachers about monitoring strategies. Metacognitive monitoring skills (such as the ones outlined in this study) are not specifically defined in curriculum documents and hence teachers are not required to teach them. Research has shown that while teachers spend time during lessons scaffolding cognitive strategies (such as how to summarize information), no time is usually allocated to teaching monitoring strategies and how this can be applied to learning (Azevedo, 2005). Given that research indicates students need to be explicitly taught SRL strategies (Dignath et al., 2008; Donker et al., 2014; Hattie et al., 1996), it is therefore not surprising that young students may lack experience with the strategies (Azevedo et al., 2010), and have inadequate language to verbalize them (Pekrun, 2020). The implication from these findings highlight a need for policy to specifically incorporate monitoring capabilities in syllabus and curriculum documents, not only as it relates to technology, but also more generally.

Conversely, some SRL strategies *are* specifically outlined in curriculum documents and therefore explicitly taught in elementary school classrooms (for example: *planning* when writing (*Planning*), *evaluating adequacy of [online] content* (*Monitoring*), *Cognitive Strategies* such as *selecting information sources*, *reading*, *summarizing* and *reviewing,* as well as controlling and interacting with the digital device). In our study, students were able to demonstrate and verbalize known cognitive strategies to locate, organize and summarize information in the hypermedia environment, as they possessed both the knowledge and language of these strategies. Additionally, students were confident to monitor the content they had chosen, supported by knowledge (and therefore language) and explained through the 'talk-aloud' protocol. Students demonstrated the ability to self-regulate their digital device (*control of context* within the category *Processes for Handling Task Difficulties and Demands)* and were competently able to utilise the affordances of their device, exhibiting skills such as changing volume, pinching the screen to manipulate size, navigating videos, screenshot and cropping manipulation of text and photos, using drawing tools and including emojis in written work to enhance meaning. These findings suggest that elementary students are able to navigate complex, open-ended, non-linear hypermedia environments (Azevedo et al., 2013). The expectation to integrate technology into the elementary classroom is clearly outlined in policy and syllabus documents (see NSW Education Standards Authority, 2021) and this was confidently demonstrated by our participants. However, planning skills for writing tasks were not automatically exhibited by students. Overall, students were able to complete a research task in a hypermedia environment, despite limited use of planning and monitoring processes. However, given the extant research on the improvement of academic achievement with metacognitive strategy use (Azevedo, 2005; Dignath & Veenman, 2021; Muijs & Bokhove, 2020), the true academic potential of our participants is therefore unknown.

### Limitations and future research

Using a talk-aloud protocol was a new experience for these participants. As such, some students found it challenging to verbalize their thinking processes (Pekrun, 2020) and instead opted to describe the content they were interacting with, rather than their judgements around the content. It was evident students did not have the language to be able to describe their thinking processes. Azevedo and colleagues acknowledge this limitation in their previous studies (e.g., Azevedo et al., 2010), noting that the complex nature of self-regulation makes it important to determine whether students possess the metacognitive knowledge but are unable to access or use that knowledge. Being able to prompt or guide students could have possibly encouraged more self-regulatory behavior being exhibited and could be considered in future studies.

Assigning valances to self-regulatory behavior would provide additional information about how successfully a student was able to use the strategy (Azevedo et al., 2010). For example, when a student demonstrated a *judgement of learning* by verbalising that they understood the information, researchers could determine whether the student made an accurate judgement by assigning either a positive ( +) (*yes, they do understand*) or negative (-) (*no, they do not understand*) valance to the behavior. Young students are not necessarily accurate with their metacognitive thinking as they are developing their self-regulatory capability (Pekrun, 2020). Specific training and guidance from teachers is strongly recommended in order to develop effective metacognitive knowledge and ability in young students (Schneider, 2008).

The purposeful sampling method utilized in this study is acknowledged as a limitation for the generalizability of results. However, our sample size aligns with mixed methods research processes (Creswell and Creswell, 2017; Greene, 2007), where the depth of both quantitative and qualitative data analysis impacts the number of participants included in the study. While this sample was confined to one cohort, the depth of analysis that was performed and the systematic nature of that analysis is unique in the field and provides insights into the nature of self-regulation amongst elementary students working in digital environments. Considering context, teacher time and school restraints, this study is comparable to previous studies using this coding scheme (Azevedo et al., 2003 (*n* = 51); Azevedo et al., 2007 (*n* = 82)). Further research involving other cohorts of students and also larger sample sizes may offer additional insights into reliability and generalizability of elementary school students' self-regulation when using technology.

## Conclusion

This study provides convincing evidence to describe and explain the SRL processes elementary students use while learning independently in a hypermedia environment. Ultimately, our findings show that some SRL strategies that are outlined in curriculum documents, such as cognitive strategies to read, re-read and summarize information, evaluating the online content, and control of their digital device and context, were known, demonstrated and verbalized by students. However, planning and goal-setting skills were not automatically utilized by all participants in this study despite the inclusion of these skills in the curriculum. The SRL strategies that are not clearly defined in policy or curriculum, in particular specific monitoring of learning strategies (for example *feeling of knowing, judgement of learning, self-questioning* and *monitoring progress towards goals*) were rarely used or acknowledged verbally. Students need to deploy several metacognitive processes in order to make decisions about their plans, goals, strategies and efforts within complex digital contexts (Azevedo et al., 2013). These metacognitive planning and monitoring strategies can be explicitly taught to students (Dignath & Veenman, 2021; Schneider, 2008) and are crucial for effective learning in hypermedia environments so that students are able to independently monitor, modify and adapt their processes as necessary in order to meet learning requirements or as contextual conditions change (Azevedo et al., 2010, 2013). This study highlights that young students therefore need explicit teaching of the five key components of SRL required in digital contexts: *Planning, Monitoring, Strategy Use, Processes for Handling Task Demands and Difficulties* and *Interest* (Azevedo et al., 2004). Language to describe, explain and support their self-regulatory process or strategy will enable them to verbalize their processes (Zimmerman & Schunk, 2011), which is a defining attribute of being a self-regulated learner.

The implications from this study are apparent: if students are to become independent self-regulated learners, with known benefits including increased academic performance, engagement, and motivation (Dignath & Veenman, 2021; Muijs & Bokhove, 2020), then all five areas of SRL (*Planning, Monitoring, Strategy Use, Processes for Handing Task Demands and Difficulties and Interest*) need to be included in policy and curriculum documents before they can be known and hence explicitly taught by teachers. Therefore, policy and practice considerations resulting from this study include: updating of policy and curriculum to specifically outline monitoring strategies to equip teachers in designing evidence-based scaffolds for their students while using hypermedia, information for software designers regarding the SRL processes that require support for elementary students within programs, professional learning for teachers about SRL strategies in digital contexts and a better understanding of SRL strategies incorporated into initial teacher education programs, promoting its importance for life-long learning success. In order to develop as self-regulated learners, particularly in situations such as COVID-19 where students may be required to learn remotely, it is imperative that elementary students are explicitly taught all five key components of SRL for use in computer-based learning environments.

## Data Availability

The data that support the findings of this study are available from the corresponding author upon reasonable request.

## Appendix A

Azevedo, Cromley & Siebert's (2004) coding framework with updated elementary school student examples.

**Table:**

	**Azevedo coding plan (Class & variable) (2004)**	**Description (from **Azevedo et al, 2004**)**	**Examples from participants in this study**
**1) PLANNING**	**- Planning**	*'A plan involves coordinating the selection of operators. Its execution involves making behavior conditional on the state of the problem and a hierarchy of goals and sub-goals.'*	(ID47) I first just read stuff through and find out what, like, I could research and focus on. 'Cause sometimes I'm not sure at the beginning what I want to choose. So…might just read through this part, just quickly
	**- Goal setting**	*'Consistent either of operations that are possible, postponed, or intended, or of states that are expected to be obtained. Goals can be identified because they have no reference to already-existing states.'*	(ID27) (space exploration – after reading for 3 min) I'm gonna start my blurb by who was the first person and how he travelled and what he travelled in
	**- Prior knowledge activation**	*'Searching memory for relevant prior knowledge either before beginning performance of a task or during task performance.'*	(ID50) I'm writing about what I've heard about Black Holes (ID67) (talking about constellations)…everyday objects—like the saucepan, 'cause I can see that from my house too
	**- Recycle goal in working memory**	*'Restating the goal (e.g., question or parts of a question) in working memory (WM)'*	(ID38) Oooo, something about the Milky Way, that interests me! I wanna see if it would interest someone else if I was sending it to someone (ID83) Um, I've just found some more information that I think would be relevant for people to know.
**2) MONITORING**	**- judgement of learning**	*'Learner becomes aware that they don't know or understand everything they read.'*	(ID02)I think it's [the video] very informative, but I don't get, I don't get anything of it because it's probably not made for my…and I've never heard of those words (ID76) That paragraph is a little bit too hard for me to comprehend….. I try and read it again and if I don't get it, I'll just go past it
	**- feeling of knowing**	*'Learner is aware of having read something in the past and having some understanding of it, but not being able to recall it on demand.'*	(ID53) *(after typing down some information)* Oh I actually kind of made a mistake, I'm pretty sure, I'm pretty sure, it's from scientists instead but…um…but yeah.* (goes back to webpage to check)* (*Researcher*) Is this more info that you know?* (student trying to type info from memory)* (ID67) Yes, but I can't remember it
	**- self-questioning**	*'Posing a question and re-reading to improve understanding of the content.'*	(ID69) *(reading through information)* OK, should I use that? (ID37) *(while viewing photo of black hole)* Dunno why they're orange?
	**- content evaluation**	*'Monitoring content relative to goals.'*	(ID27) Now I've realised I should probably change and make it the nationalities of the person. So they understand more of them (ID47) So I've basically finished those last three paragraphs so I'm just going to see if there's any more information that I've missed up the top here
	**-identify adequacy of information**	*'Assessing the usefulness and/or adequacy of the content (reading, watching, *etc*.)'*	(ID10) I'm going to write this important piece of information (ID67) Ooo! Here we go (ID83) Um, I've just found some more information that I think would be relevant for people to know…about the surface on Venus. Because I had written down what it looks like and now I'm figuring out why. And I think that that would be something that is important to know
	**- monitor progress towards goals**	*'Assessing whether previously set goal has been met.'*	(ID11) Well…I've written two sentences and they kind of explain what I've learnt from that video, um, but I need…I think I might need more information, so I'm gonna go back. I'm gonna actually read the article to see …what it tells me (ID83) I'm thinking now to move on from the way and its orbits and rotations now, because I've gotten enough information on that
**3) STRATEGY USE**	**- selecting new informational source**	*'The selection and use of various cognitive strategies for memory, learning, reasoning, problem solving and thinking. May include selecting a new representation, coordinating multiple representations, *etc*.'*	*[student reads about space and then switches to watching a video about it.]* (ID02) I'm going to watch this video
	**- coordinating informational sources**	*'Coordinating multiple representations, e.g., drawing and notes'*	*[copying a picture from Britannica]* *(Researcher) What are you doing now?* (ID03) I'm gonna add it to the..um…the research
	**- read new paragraph**	*'The selection and use of a paragraph different from the one the student was reading.'*	(ID27) Now I'm gonna read a little bit more of the article, because I finished my first two sentences
	**- review notes**	*'Reviewing learner's notes.'*	(ID76) Now, I'm just gonna go over what I've already got—see if everything written just makes sense
	**- memorization**	*'Learner tries to memorize text, diagram, *etc*.'*	*(Researcher) What are you thinking?* (ID80) Um…just..really just looking at it, getting…trying to memorize what I need to know, writing it in my own words and then just… I really just keep going back until, like, it's done. I might do some points as well
	**- free search**	*'Searching the hypermedia environment without specifying a specific plan or goal.'*	*(There were no examples of this from any participants.)*
	**- goal-directed search**	*'Searching the hypermedia environment after specifying a specific plan or goal.'*	(ID76) I'm gonna search up how many Black Holes the scientists think there is in the Milky Way (ID37) I couldn't find what I needed, so I'm searching it up on Safari
	**- summarization**	*'Summarizing what was just read, inspected, or heard in the hypermedia environment.'*	(ID02) So I'm choosing my own language rather than copying this whole sentence down (ID27) So, whenever I read an article and I finish, I try to think of how I can make it my own words, so I don't copy it
	**- taking notes**	*'Copying text from the hypermedia environment.'*	(ID63) This paragraph looks interesting to me, so I'm gonna copy it down
	**- drawing**	*'Making a drawing or diagram to assist in learning.'*	(ID30) I want to draw a black hole…on the Notes I think. *(Researcher): Why is that?* (ID30) Because…*(long pause)* *(Researcher) Do you know there's no right or wrong—what's going on in your head with that? Why are you…do you want to draw it?* (ID30) 'Cause I want…I want to see if I can, 'cause I, I like drawing. Also because it's sometimes you can't get….aaa..like it….sometimes people like to draw instead of writing
	**- re-reading**	*'Re-reading or revisiting a section of the hypermedia environment.'*	(ID02) I'm thinking I'm just reading again…just reading the paragraph to see if there's any good things in here (ID24) I'm going back 'cause I can't remember what the fact was
	**- inferences**	*'Making inferences based on what was read, seen, or heard in the hypermedia environment.'*	(ID 76) I'm thinking that it's really scary that in the whole Milky Way system there is a hundred million Black Holes left, from stars (ID13) I never knew black holes were that big!
	**- hypothesizing**	*'Asking questions that go beyond what was read, seen or heard.'*	(ID47) *(after reading) *It says here that they purposely crashed one of their crafts into the moon to search for water, and that they couldn't find any. And I'm wondering if they have continued to look for some or if they just completely just based it off that one…aircraft? (ID37) I'm just thinking *(about what they’d just read about black holes)*: What happens if a human goes near it? Or some kind of spacecraft?
	**- knowledge elaboration**	*'Elaborating on what was just read, seen, or heard with prior knowledge.'*	(ID10) Black Holes actually do exist and it's kind of scary because you know your planet might be sucked in to a Black Hole at any moment! (ID22) It's crazy to think that, um, it's coming from 3.8 billion light years away…
	**- mnemonic**	*'Using a verbal or visual memory technique to remember content.'*	*(There were no examples of this from any participants.)*
	**- evaluate content as answer to goal**	*'Statement that what was just read and/or seen meets a goal or sub-goal.'*	(ID64) Oh! Ok. Well it just answered my question!
	**- find location in environment**	*'Statement about where in environment learner had been reading.'*	(ID69) *(scrolling through previously read text)* Click on the link. Where is it? Ah, that one. *(Clicks on link)* (ID25) *(after deciding he needs more information for his blurb)* …I'm gonna start back where I was…which was *[scrolling]*…..um, over here
	****(new)Watching video for information**		(ID02) I'm going to watch this video
	****(new) typing using prior knowledge no hypermedia**		(ID51) Some of this stuff I've read, some of this stuff I've known, like the meteorites
**4) PROCESSES FOR HANDLING TASK DIFFICULTIES AND DEMANDS**	**- time and effort planning**	*'Attempts to intentionally control behavior.'*	(ID77) How much longer do I have?
	**- help-seeking behavior**	*'Learner seeks assistance regarding either the adequateness of their answer or their instructional behavior.'*	(ID76) What does ‘density’ mean? (ID77) How long does it have to be?
	**- task difficulty**	*Learner indicates one of the following: (1) the task is either easy or difficult, (2) the questions are either simple or difficult, (3) using the hypermedia environment is more difficult than using a book*	(ID07) *(when asked what he was thinking)* I don't really know how to spell. So….[long pause] I keep on getting lost (ID30) It's kind of hard ‘cause…..*(unfinished) (he couldn’t find the answer to his questions, yet didn’t know what to do to do about it)*
	**- control of context**	*Using features of the hypermedia environment to enhance the reading and viewing of information*	(ID63) Now I'm going back onto Notes, I'm going to paste that down and edit it (ID10) *pinched screen to zoom to enlarge webpage* (ID02) So, I've split the screen, so I can read and type at the same time. And so I can also make my own words instead of having to go in and out of stuff (ID77) I usually put, like, emojis if I'm writing on, like, Notes or like one of them, just to, like um, so I can go back and see…what I've written there. So like here (points to star emoji) I've talked about stars and here (points to other emoji) I've talked about constellations. So, that's what I usually do (ID22) *paused video, pinched screen to zoom in on still picture to have a closer look*
	**- expectation of adequacy of information**	*'Expecting that a certain type of representation will prove adequate given the current goal.'*	(ID11) There's another video and it might tell me something else, so that's why I want to watch that as well
**5) MOTIVATION**	**- interest statement**	*(No definition provided)*	(ID02) I chose to write this because it is an interesting fact, and it might 'wow' people (ID27) I really like space, and I'm trying to be an aerospace engineer (ID13) *(after watching video)* That was very interesting!

## *T*-test results for categories

### Paired Sample T-Test results for the 'Planning' variables

**Table:**

	Planning	Goal setting	Prior knowledge activation	Recycle goal in working memory
Planning	-			
Goal setting	6.571***	-		
Prior knowledge activation	-0.741	-2.089*	-	
Recycle goal in working memory	9.467***	4.392***	2.653**	-

For all *t*-tests, degrees of freedom = 47.

**p* < 0.05, ***p* < 0.01, ****p* < 0.001.

### Paired Sample T-Test results for the 'Monitoring' variables

**Table:**

	Judgement of learning	Feeling of knowing	Self-questioning	Content evaluation	Identify adequacy of information	Monitor progress towards goals
Judgement of learning	-					
Feeling of knowing	3.653***	-				
Self-questioning	0.858	-2.677**	-			
Content evaluation	-1.979	-4.902***	-2.405**	-		
Identify adequacy of information	-0.948	-4.172***	-1.772	0.992	-	
Monitor progress towards goals	0.211	-2.468**	-0.527	1.945	1.077	-

For all *t*-tests, degrees of freedom = 47.

**p* < 0.05, ***p* < 0.01, ****p* < 0.001.

### Paired Sample T-Test results for the 'Processes for Handling Task Difficulties and Demands' variables

**Table:**

	time and effort planning	help-seeking behavior	task difficulty	control of context	expectation of adequacy of information
time and effort planning	-				
help-seeking behavior	0.682	-			
task difficulty	1.286	1.103	-		
control of context	-6.397***	-6.483***	-6.781***	-	
expectation of adequacy of information	0.842	0.256	-0.851	6.51***	-

For all *t*-tests, degrees of freedom = 47.

**p* < 0.05, ***p* < 0.01, ****p* < 0.001.
